# TGF-β1 promotes SCD1 expression via the PI3K-Akt-mTOR-SREBP1 signaling pathway in lung fibroblasts

**DOI:** 10.1186/s12931-023-02313-9

**Published:** 2023-01-10

**Authors:** Zili Zhou, Shixiu Liang, Zicong Zhou, Jieyi Liu, Jinming Zhang, Xiaojing Meng, Fei Zou, Haijin Zhao, Changhui Yu, Shaoxi Cai

**Affiliations:** 1 Chronic Airways Diseases Laboratory, Department of Respiratory and Critical Care Medicine, Nanfang Hospital, Southern Medical University, Guangzhou, 510515 China; 2 Guangdong Provincial Key Laboratory of Tropical Disease Research, Department of Occupational Health and Medicine, School of Public Health, Southern Medical University, Guangzhou, 510515 China

**Keywords:** House dust mite, Airway remodeling, Fibroblasts, SCD1, SREBP1

## Abstract

**Background:**

Lung fibroblast activation is associated with airway remodeling during asthma progression. Stearoyl-CoA desaturase 1 (SCD1) plays an important role in the response of fibroblasts to growth factors. This study aimed to explore the effects of SCD1 on fibroblast activation induced by transforming growth factor-β1 (TGF-β1) and the role of the phosphatidylinositol-3-kinase-AKT serine-threonine protein kinase-mechanistic target of rapamycin (PI3K-Akt-mTOR) pathway on the regulation of SCD1 expression in airway remodeling.

**Methods:**

Female C57BL/6 mice were sensitized and challenged with house dust mites to generate a chronic asthma model. The inhibitor of SCD1 was injected i.g. before each challenge. The airway hyper-responsiveness to methacholine was evaluated, and airway remodeling and airway inflammation were assessed by histology. The effects of SCD1 on fibroblast activation were evaluated in vitro using an SCD1 inhibitor and oleic acid and via the knockdown of SCD1. The involvement of the PI3K-Akt-mTOR-sterol regulatory element-binding protein 1 (SREBP1) pathway in lung fibroblasts was investigated using relevant inhibitors.

**Results:**

The expression of SCD1 was increased in fibroblasts exposed to TGF-β1. The inhibition of SCD1 markedly ameliorated airway remodeling and lung fibroblast activation in peripheral airways. The knockdown or inhibition of SCD1 resulted in significantly reduced extracellular matrix production in TGF-β1-treated fibroblasts, but this effect was reversed by the addition of exogenous oleic acid. The PI3K-Akt-mTOR-SREBP1 pathway was found to be involved in the regulation of SCD1 expression and lung fibroblast activation.

**Conclusions:**

The data obtained in this study indicate that SCD1 expression contributes to fibroblast activation and airway remodeling and that the inhibition of SCD1 may be a therapeutic strategy for airway remodeling in asthma.

**Supplementary Information:**

The online version contains supplementary material available at 10.1186/s12931-023-02313-9.

## Background

Asthma is a chronic respiratory disease characterized by reversible airflow obstruction, chronic inflammation, airway remodeling, and airway hyper-responsiveness (AHR) [1]. Airway remodeling occurs during disease progression and leads to chronic structural changes, including epithelial shedding, goblet cell hyperplasia, airway smooth muscle hypertrophy, basement membrane thickening, and subepithelial fibrosis [2]. Upon activation, lung fibroblasts express the cytoskeletal protein alpha-smooth muscle actin (α-SMA) and produce chemokines and extracellular matrix (ECM) proteins, including fibronectins and collagens, which leads to subepithelial fibrosis [3]. Excessive deposition of ECM proteins is a hallmark of chronic asthma and causes airway lumen stiffening and narrowing in patients with asthma [4, 5]. Transforming growth factor-β (TGF-β) is an important mediator that activates fibroblasts [6]. Increased levels of TGF-β1 have been observed in bronchoalveolar lavage fluid (BALF) from asthmatic patients [7].

Stearoyl-CoA desaturase 1 (SCD1) is a rate-limiting enzyme responsible for de novo lipogenesis that is anchored in the endoplasmic reticulum membrane and catalyzes the formation of monounsaturated fatty acids (MUFAs) from saturated fatty acids (SFAs) [8]. The expression of SCD1 is decreased in bronchial epithelial cells from patients with asthma [9]. However, the effects of SCD1 on fibroblast activation in airway remodeling remain unclear.

Sterol regulatory element-binding protein 1 (SREBP1) is a key transcriptional regulator of enzymes involved in fatty acid metabolism [10]. After cleavage in the endoplasmic reticulum, SREBP1 enters the nucleus and binds to a specific DNA sequence to activate the transcription of the lipogenic genes acetyl-CoA carboxylase alpha (ACACA), fatty acid synthase (FASN) and SCD1 [11]. The available data increasingly demonstrates that the mechanistic target of rapamycin (mTOR) is responsible for the activation of SREBP1 and lipid synthesis in the presence of cell differentiation and proliferation factors [12, 13].

In this study, we demonstrated that TGF-β1 promoted SCD1 expression in lung fibroblasts. The inhibition or knockdown of SCD1 not only suppressed myofibroblast differentiation but also attenuated airway remodeling, and these effects were alleviated by the addition of exogenous oleic acid (OA). Moreover, TGF-β1 promoted SCD1 expression via the PI3K-Akt-mTOR-SREBP1 signaling. Our results suggest that SCD1 is essential for fibroblast activation in response to TGF-β1 and indicate a novel therapeutic target in the treatment of airway remodeling in asthma.

## Materials and methods

### Reagents

House dust mite (HDM) extract was purchased from Greer laboratories (Lenoir, NC, USA). Enzyme-linked immunosorbent assay (ELISA) kits for immunoglobulin E (IgE) were purchased from RayBiotech (Norcross, GA, USA). Recombinant human TGF-β1 was purchased from R&D Systems (Minneapolis, MN, USA). The SCD1 inhibitor A939572 and the PI3K inhibitor LY294002 were purchased from MedChemExpress (Shanghai, China). The SREBP1 inhibitor Fatostatin HBr and the mTOR inhibitor Torin1 were purchased from Selleck (Shanghai, China). OA-bovine serum albumin (BSA) was purchased from Sigma‒Aldrich (St. Louis, MO, USA). Small interfering RNA targeting SCD1 (siSCD1) and negative control small interfering RNA (siNC) were obtained from GenePharma (Suzhou, China). The siRNA sequence information is listed in Additional file 1: Table S1. The primers used in this study were synthesized by Synbio Technologies (Suzhou, China). Cytosolic, nuclear and membrane proteins were extracted using a Minute™ Plasma Membrane Protein Isolation and Cell Fractionation Kit (Invent Biotechnologies, Beijing, China) according to the manufacturer’s instructions. The antibodies used are listed in Additional file 1: Table S2.

### Cell culture, inhibitor treatment, and transfection

The human fetal lung fibroblast cell line (HFL1) was obtained from our laboratory as previously described [14]. HFL1 cells were cultured in F12K medium supplemented with 10% fetal bovine serum (Gibco, New Zealand) at 37 °C in an atmosphere with 5% CO_2_. Once the HFL1 cells reached 80–90% confluence, they were placed in serum-free medium for 24 h and then treated with inhibitors and TGF-β1 for the indicated times. siRNA transfection was performed with a transfection reagent (Lipofectamine 3000 from Invitrogen) according to the manufacturer’s protocol.

### Animals and HDM-induced chronic asthma model

Female C57BL/6 mice aged 6–8 weeks were obtained from Southern Medical University and housed under pathogen-free conditions with 12 h light/dark cycles. The mice were acclimatized for one week before the start of the experiments. All animal studies were conducted in accordance with the animal use guidelines of the Southern Medical University, and the protocols were approved by the Animal Ethics Committee of Southern Medical University. A total of 40 mice were used (n = 10 in each experimental group). The mice were randomly divided into four groups: (1) control group, phosphate-buffered saline (PBS)-sensitized/PBS-challenged mice treated with the solvent; (2) HDM group, HDM-sensitized/HDM-challenged mice treated with the solvent; (3) A93 group, PBS-sensitized/PBS-challenged mice treated with A939572; and (4) HDM + A93 group, HDM-sensitized/HDM-challenged mice treated with A939572.

Briefly, the mice were sensitized with 25 μg of HDM extract diluted in 100 μl of PBS on Days 1 and 8 via intraperitoneal injections. Beginning on Day 15, the mice were exposed intranasally to 25 μg of HDM extract (in 20 μl of PBS) for 5 consecutive days and then allowed to rest for 2 days, and this cycle was repeated for 5 weeks. The control mice were sensitized or challenged with the same volume of PBS. The mice were gavaged with A939572 (5 mg/kg) 2 h before challenge, and the control mice received the same volume of solvent for comparison. The mice were sacrificed 24 h after the last HDM or PBS challenge.

### Assessment of AHR

The lung resistance index of anesthetized and mechanically ventilated (Buxco Electronics, Wilmington, NC, USA) mice was determined in response to increasing doses of methacholine (0, 3.125, 6.25, 12.5, 25, and 50 mg/ml) administered by ultrasonic nebulization. Measurements of lung resistance were performed every 5 min following each nebulization step until a plateau phase was reached.

### Serum total IgE

Blood samples were collected from the retro-orbital plexus, incubated at room temperature for 1 h, and then centrifuged at 3000 rpm for 10 min. The supernatants were harvested, and the total IgE level was measured using an ELISA kit according to the manufacturer’s instructions.

### Collection of bronchoalveolar lavage fluid (BALF)

BALF was collected approximately 24 h after the last HDM exposure. Briefly, the mice were sacrificed by exsanguination. Then, the lungs were lavaged using a cannula inserted in the trachea, and the lungs were instilled with 1 ml of PBS. After a tenfold dilution, the total cells in BALF were counted using a cell counting plate. The cells were centrifuged at 1500 rpm for 5 min and resuspended in 40 μl of 4% paraformaldehyde. Smears were prepared by dropping the cell suspension onto poly-L-lysine coated slides. After staining with Wright-Giemsa staining, the numbers of differential inflammatory cells in 200 cells of BALF were counted under a microscope in a blinded manner.

### Histology

The left lung lobes were fixed in 4% neutral paraformaldehyde and then embedded in paraffin according to standard procedures. The right lung lobes were immediately snap-frozen in liquid nitrogen and stored at −80 °C for subsequent protein or RNA analysis. Lung sections (4 μm) were used for hematoxylin and eosin (HE) staining. The results were scored by three observers in a random blinded manner and semiquantified as previously described [15]. Periodic acid-Schiff (PAS) staining was performed to quantify the percentages of goblet cells among airway epithelial cells as previously described [16]. Peribronchial collagen deposition was assessed by Masson trichrome staining, and quantified using Image-Pro Plus 6.0 software (Media Cybernetics, Rockville, MD, USA) as previously described [17].

### Immunohistochemistry (IHC)

Sections of lung tissue were treated with 0.3% H_2_O_2_ for 10 min to quench endogenous peroxidase activity and then blocked in PBS containing 5% BSA for 30 min. After incubation with rabbit anti-collagen I antibody (ab34710, Abcam, USA) at a dilution of 1:200 overnight at 4 °C, the sections were incubated with biotinylated anti-rabbit IgG secondary antibody (Zsbio, Beijing, China) for 1 h and exposed to a substrate chromogen mixture for 2 min (Zsbio, Beijing, China). The staining intensity of collagen I per micrometer length of the basement membrane of bronchioles was calculated using Image-Pro Plus 6.0 software as previously described [18].

### Western blotting

After treatments, lung tissue or cells were lysed in lysis buffer containing PMSF, protease and phosphatase inhibitors (Keygen Biotech, Nanjing, China). The lysates were separated on SDS‒PAGE gels, transferred to PVDF membranes (Millipore, Bedford, MA, USA), and then immunoblotted with primary antibodies overnight at 4 °C. All antibodies were diluted 1:1000, with the exception of Lamin B1, which was diluted 1:10,000. After incubation with secondary antibodies conjugated with IRDye® 680 (LI-COR Biosciences, Lincoln, NE, USA) at a dilution of 1:10,000 for 1 h at room temperature, immunoreactive bands were detected using an Odyssey imaging system (LI-COR Biosciences, Lincoln, NE, USA). The quantitative analysis was performed using ImageJ 1.8.0 software (NIH, Bethesda, MD, USA).

### RNA isolation and quantitative real-time polymerase chain reaction (qPCR) analysis

RNA was isolated from cells or lung tissue using an RNAiso Plus Kit (Takara, Dalian, China) according to the manufacturer’s instructions. Reverse transcription was performed with reverse transcription reagents (Takara, Dalian, China). The levels of mRNA were measured with a Bio-Rad CFX96 Real-Time system using Hieff® qPCR SYBR® Green Master Mix (Yeasen Biotech, Shanghai, China). The relative changes in mRNA expression were quantified using the 2^−ΔΔCq^ method. The primers used in the present study are listed in Additional file 1: Table S3.

### Immunofluorescence staining

Lung tissue was fixed in 4% neutral paraformaldehyde at 4 °C for 24 h, treated with 30% sucrose at 4 °C for 24 h, embedded in O.C.T. compound (Sakura Finetek, Torrance, CA, USA) and used to prepare lung sectsions (6 μm). HFL1 cells were seeded on glass-cover dishes. HFL1 cells were washed with PBS after being treated and then fixed in 4% neutral paraformaldehyde. Subsequently, the lung sections or cells were treated with 0.5% Triton-X-100 (Sigma‒Aldrich) in PBS for 20 min and blocked with 5% BSA in PBS for 30 min. The samples were then incubated with primary antibodies at a dilution of 1:200 overnight at 4 °C and then with secondary antibodies conjugated with Alexa Fluor® 488 or Alexa Fluor® 594 (Invitrogen, Carlsbad, CA, USA) at a dilution of 1:100 for 1 h at room temperature. The cell nuclei were labeled with DAPI (Beyotime Biotechnology, Shanghai, China) for 5 min. Fluorescent images were captured with a laser scanning confocal microscope (Olympus, Tokyo, Japan).

### Statistical analysis

All results are presented as the means ± SEMs. The data were analyzed using GraphPad Prism 8.2.1 software (GraphPad Software, La Jolla, CA, USA). A value of *P* < 0.05 was considered to indicate statistical significance. At least three independent experiments were performed.

## Results

### TGF-β1 increases SCD1 expression in fibroblasts

**Fig. 1:**
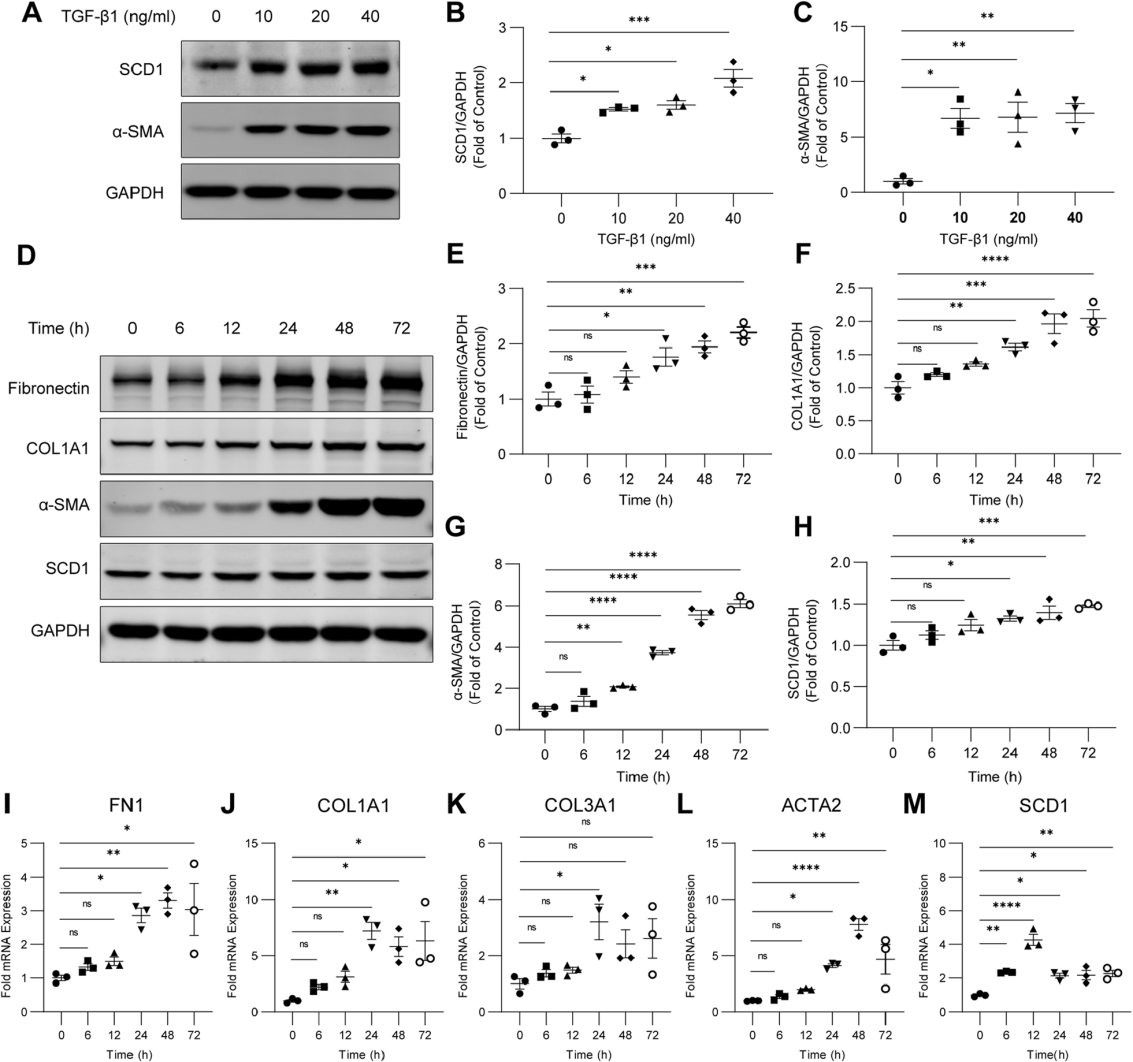
TGF-β1 increases SCD1 expression in fibroblasts. **A**–**C** HFL1 cells were treated with increasing concentrations of TGF-β1 for 24 h. The protein levels of SCD1, α-SMA, and a loading control (GAPDH) were measured by western blotting. The relative changes in the densities were detected. **D**–**H** HFL1 cells were treated with 10 ng/ml TGF-β1 for the indicated times. The protein levels of fibronectin, COL1A1, α-SMA, and SCD1 were measured by western blotting. The relative changes in the densities were detected. **I**–**M** HFL1 cells were treated with 10 ng/ml TGF-β1 for the indicated times. qPCR was performed to measure the mRNA levels of FN1, COL1A1, COL3A1, ACTA2 and SCD1. The data are representative of three independent experiments and are presented as the means ± SEMs. *P < 0.05, **P < 0.01, ***P < 0.001, and ****P < 0.0001, as determined by one-way ANOVA with the Tukey‒Kramer post hoc test

To investigate the expression of SCD1 during fibroblast activation, we examined the effects of TGF-β1 on the HFL1 cell line in vitro. We observed that TGF-β1 treatment increased SCD1 expression in a concentration-dependent manner, and the expression of α-SMA was also increased (Fig. 1A–C). In addition, western blotting demonstrated that TGF-β1 induced fibronectin, COL1A1, α-SMA and SCD1 protein expression in HFL1 cells in a time-dependent manner (Fig. 1D–H). Similarly, TGF-β1 increased the mRNA expression levels of fibronectin 1 (FN1), COL1A1, collagen type III alpha 1 (COL3A1), ACTA2 (α-SMA) and SCD1 in HFL1 cells (Fig. 1I–M). These findings suggest that TGF-β1-induced fibroblast activation is associated with increased levels of SCD1 in HFL1 cells.

### SCD1 inhibition ameliorates airway remodeling but not inflammation in an HDM-induced chronic asthma mouse model

**Fig. 2:**
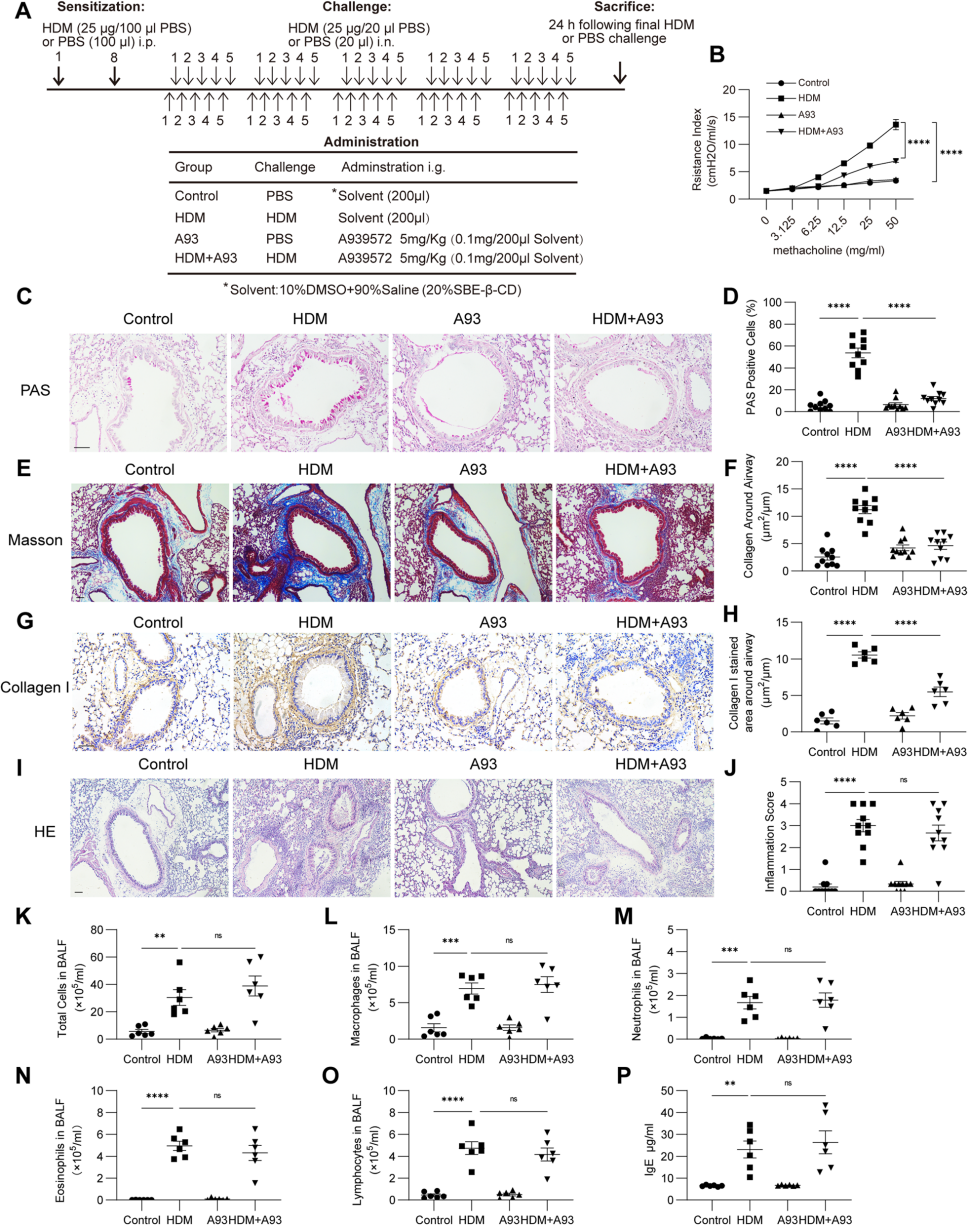
SCD1 inhibition ameliorates airway remodeling but not inflammation in an HDM-induced chronic asthma mouse model. **A** Schematic diagram of the experimental protocol for HDM sensitization and challenge. **B** AHR was measured by the lung resistance index (n = 4 mice in each group). **C**–**F** The percentages of PAS-positive airway epithelial cells and collagen around the airways were quantified (n = 10 mice in each group). **G**, **H** Representative IHC images of collagen I expression in lung sections from the different treatment groups. Scale bar, 100 μm. The collagen I-stained areas were quantified (n = 6 mice in each group). **I**, **J** Representative images of HE-stained lung tissue sections from the different treatment groups (n = 10 mice in each group). Scale bar, 100 μm. The inflammation score was determined. **K** Numbers of total cells in BALF (n = 6 mice for each group). **L**–**O** Numbers of differential inflammatory cells in BALF (n = 6 mice in each group). **P** The total serum IgE levels were assessed by ELISA (n = 6 mice in each group). The data are presented as the means ± SEMs and were statistically analyzed by one-way ANOVA with the Tukey‒Kramer posttest. *P < 0.05, **P < 0.01, ***P < 0.001, and ****P < 0.0001

To determine the effects of SCD1 on airway remodeling and airway inflammation in HDM-induced asthmatic mice, we administered A939572, a small molecule that specifically inhibits SCD1 enzymatic activity, by gavage (Fig. 2A). As expected, HDM-induced asthmatic mice exhibited a significantly increased AHR. Treatment with A939572 partially reduced AHR induced by methacholine (Fig. 2B). Overproduction of mucus and goblet cell hyperplasia were also observed after sensitization and challenge with HDM, and these effects were significantly decreased by A939572, as shown by PAS staining (Fig. 2C, D). Similarly, Masson trichrome staining showed a significant increase in collagen deposition in the interstitium of the airways and vessels of mice exposed to HDM extract, and this increase was attenuated in mice treated with A939572 (Fig. 2E, F). Moreover, the accumulation of collagen I in the peribronchiolar region induced by HDM was also reduced by treatment with A939572 (Fig. 2G, H).

One central pathway in airway remodeling involves TGF-β1-induced fibroblast activation, which leads to increased production of ECM proteins, airway wall thickening and airflow obstruction [19]. HDM exposure induced a marked elevation of TGF-β1 in BALF, and this effect was significantly decreased by treatment with A939572 (Additional file 1: Fig. S1A). Western blotting showed similar increases in the levels of the ECM proteins fibronectin and collagen type I alpha 1 (COL1A1) and the myofibroblast differentiation marker α-SMA in lung homogenates from the HDM group, and these effects were decreased by treatment with A939572 (Additional file 1: Fig. S1B). In addition, treatment with A939572 suppressed fibroblast activation, as shown by immunofluorescence colocalization of S100A4, a specific marker of fibroblasts, and fibronectin in peripheral airways (Additional file 1: Fig. S1C).

We also investigated the effects of SCD1 on airway inflammation. A histology analysis showed a typical feature of peribronchiolar and perivascular inflammatory cell infiltration in the lungs of the HDM group, but the degree of infiltration was not reduced in the mice treated with A939572 (Fig. 2I, J). Consistently, higher amounts of total cells and increased numbers of macrophages, lymphocytes, neutrophils and eosinophils in BALF were found in the mice exposed to HDM extract. We did not find a reduction in inflammatory cells in BALF of mice treated with A939572 (Fig. 2K–O). Moreover, HDM challenge increased the level of serum IgE, but treatment with A939572 did not reduce this level (Fig. 2P).

Above all, these data indicate that A939572 could relieve airway remodeling and inhibit fibroblast activation but fails to reduce airway inflammation in the HDM-induced mouse model of chronic asthma.

### SCD1 is needed for TGF-β1-induced fibroblast activation in HFL1 cells

**Fig. 3:**
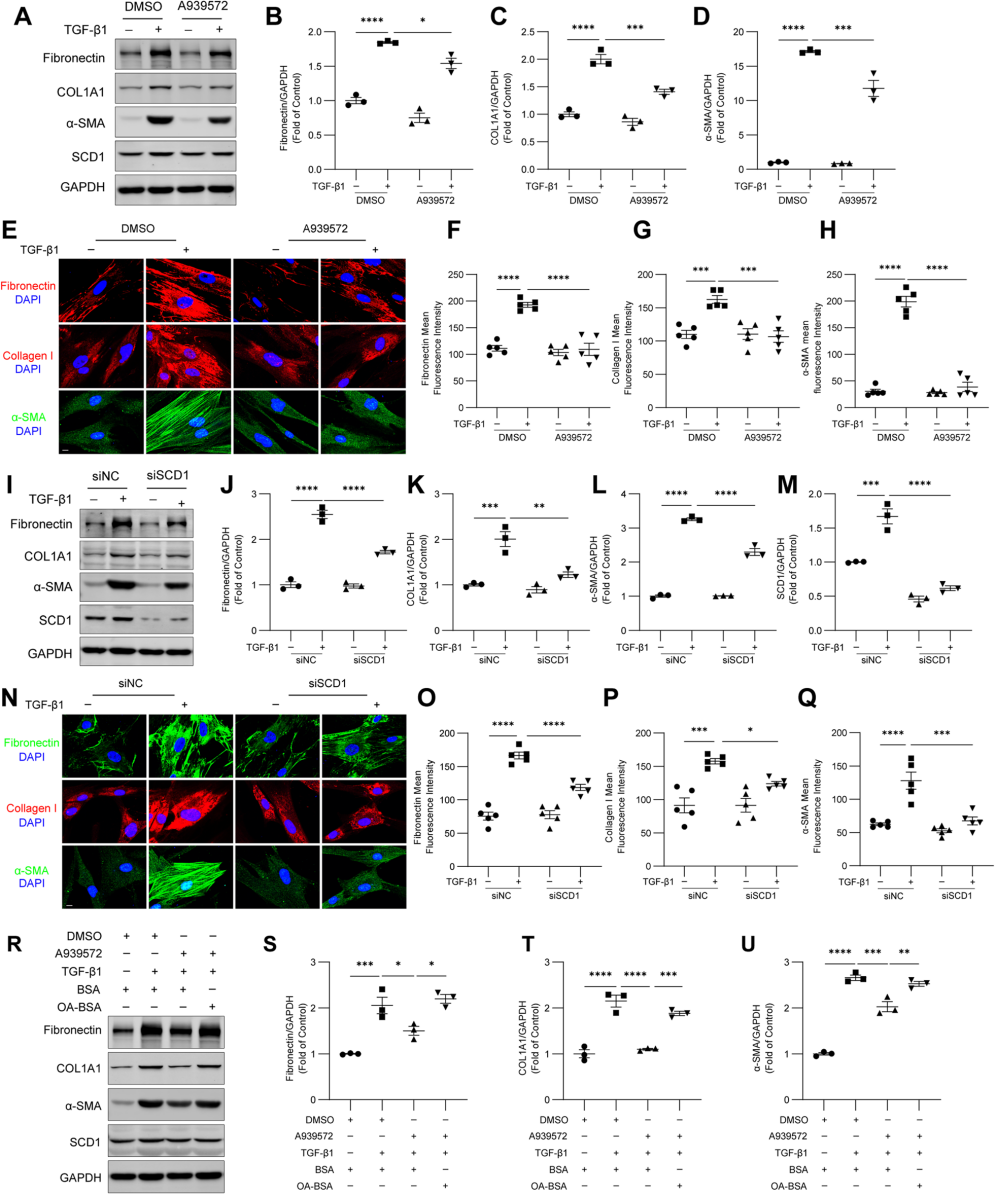
SCD1 is needed for TGF-β1-induced fibroblast activation. HFL1 cells were treated with TGF-β1 (10 ng/ml) for 24 h in the presence of A939572 (25 μM). **A**–**D** The levels of fibronectin, COL1A1, α-SMA, and SCD1 in HFL1 cells were measured by western blotting. The relative changes in band densities were quantified. **E**–**H** Immunofluorescence staining of HFL1 cells with antibodies against fibronectin, collagen I, and α-SMA was captured by confocal microscopy. Scale bar, 10 μm. The fluorescence intensities were calculated (n = 5 images from each group were used for quantification). HFL1 cells were treated with siNC or siSCD1 for 48 h and then with 10 ng/ml TGF-β1 for 24 h. **I**–**M** The levels of fibronectin, COL1A1, α-SMA, and SCD1 in HFL1 cells were measured by western blotting. The relative changes in band densities were quantified. **N**–**Q** Immunofluorescence staining of HFL1 cells with antibodies against fibronectin, collagen I and α-SMA. Scale bar, 10 μm. The fluorescence intensities were quantified (n = 5 images from each group were used for quantification). **R**–**U** HFL1 cells were treated with TGF‑β1 (10 ng/ml), A939572 (25 μM) and OA (0.3 mM) for 24 h, and the protein levels of fibronectin, COL1A1, α-SMA, and SCD1 in HFL1 cells were measured by western blotting. The relative changes in band densities were detected. The data are representative of three independent experiments and are presented as the means ± SEMs. *P < 0.05, **P < 0.01, ***P < 0.001, and ****P < 0.0001, as determined by one-way ANOVA with the Tukey‒Kramer post hoc test

We then further investigated whether SCD1 regulates TGF-β1-induced fibroblast activation. Western blotting showed that the inhibition of SCD1 partially decreased the upregulation of fibronectin, COL1A1 and α-SMA induced by TGF-β1 in HFL1 cells (Fig. 3A–D). Immunofluorescence staining of fibronectin, collagen I and α-SMA revealed a similar reduction in HFL1 cells cotreated with TGF-β1 and A939572 (Fig. 3E–H). Consistent with the results obtained with A939572 treatment, SCD1 knockdown decreased the protein expression of fibronectin, COL1A1, and α-SMA in response to TGF-β1 in HFL1 cells (Fig. 3I–M). Immunofluorescence staining showed that the fibronectin, collagen I and α-SMA levels were also reduced in HFL1 cells after SCD1 knockdown (Fig. 3N–Q).

OA is the main MUFA generated by SCD1 and is subsequently incorporated into membrane phospholipids [20]. Therefore, we used OA-BSA, a complex containing BSA that is suitable for cell culture, to investigate whether the addition of exogenous OA might rescue the inhibition of fibroblast activation induced by A939572 and SCD1 knockdown. We observed that the A939572-induced inhibition of protein production was obviously alleviated in the presence of exogenous OA (Fig. 3R–U). Moreover, treatment with exogenous OA increased the protein levels of fibronectin, COL1A1 and α-SMA in SCD1-knockdown HFL1 cells (Additional file 1: Fig. S2A–D). Collectively, these results suggest that SCD1 is needed for TGF-β1-induced fibroblast activation and ECM protein production.

### SREBP1 regulates TGF-β1-induced SCD1 expression and fibroblast activation

**Fig. 4:**
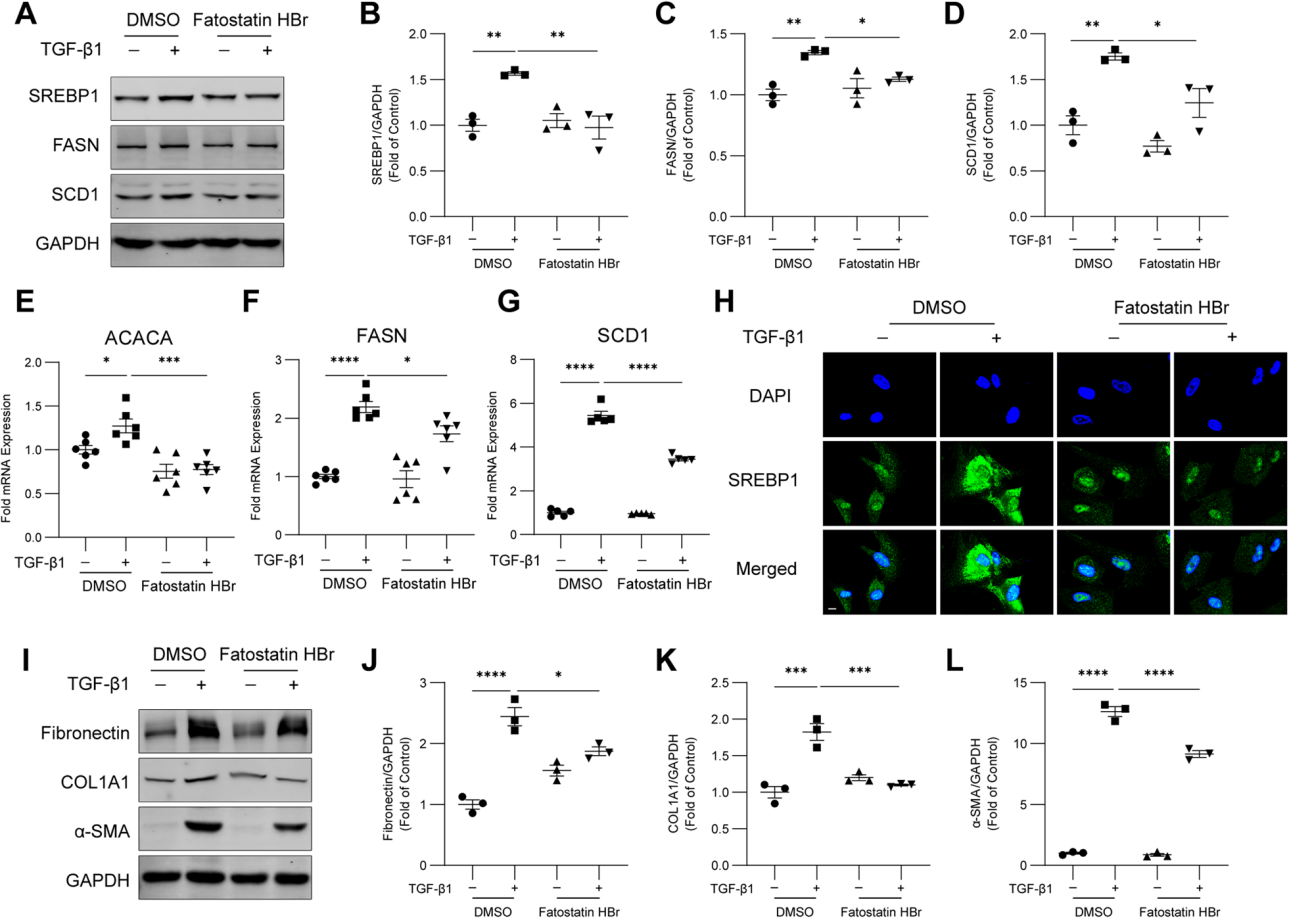
SREBP1 regulates TGF-β1-induced SCD1 expression and fibroblast activation. HFL1 cells were treated with TGF‑β1 (10 ng/ml) and Fatostatin HBr (5 µM) for 24 h. **A**–**D** A western blot analysis of SREBP1, FASN and SCD1 was performed. **E**–**G** qPCR was performed to measure the mRNA levels of ACACA, FASN, and SCD1. **H** The immunofluorescence staining of HFL1 cells with antibodies against SREBP1 was captured by confocal microscopy. Scale bar, 10 μm. **I**–**L** A western blot analysis of fibronectin, COL1A1, and α-SMA was performed. The data are representative of three or six independent experiments and are presented as the means ± SEMs. *P < 0.05, **P < 0.01, ***P < 0.001, and ****P < 0.0001, as determined by one-way ANOVA with the Tukey‒Kramer post hoc test

SREBP1 plays a transcriptional activation role in enzymes associated with fatty acid synthesis [21]. To determine whether SREBP1 regulates TGF-β1-induced SCD1 expression in HFL1 cells, we used Fatostatin HBr, a specific inhibitor of SREBP1 activation. Interestingly, treatment with Fatostatin HBr decreased the expression of SREBP1 and the downstream fatty acid synthesis enzymes FASN and SCD1 in HFL1 cells (Fig. 4A–D). Similarly, a significant reduction in the transcript levels of the SREBP1 target genes ACACA, FASN, and SCD1 was observed in HFL1 cells treated with TGF-β1 and Fatostatin HBr (Fig. 4E–G). Immunofluorescence staining also showed reduced expression of SREBP1 after treatment with Fatostatin HBr (Fig. 4H). The fibroblast activation markers fibronectin, COL1A1 and α-SMA were also decreased by treatment with Fatostatin HBr (Fig. 4I–L). These results indicate that SREBP1 contributes to SCD1 expression and fibroblast activation downstream of TGF-β1.

### TGF-β1 increases SCD1 expression and promotes fibroblast activation via PI3K-Akt-mTOR signaling

**Fig. 5:**
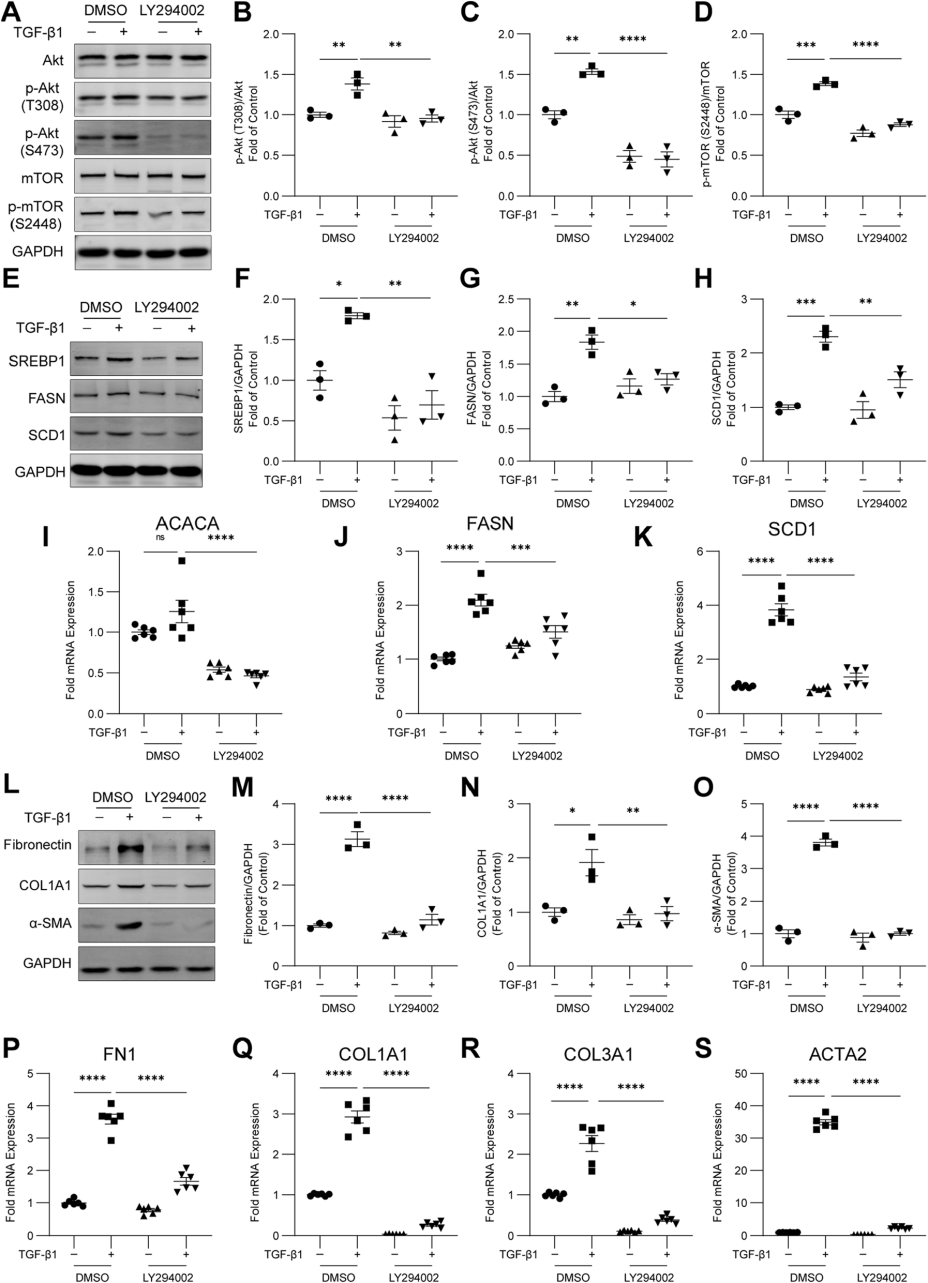
TGF-β1 increases SCD1 expression and promotes fibroblast activation via PI3K-Akt-mTOR signaling. **A**–**D** HFL1 cells were treated with TGF‑β1 (10 ng/ml) and LY294002 (10 μM) for 2 h, and a western blot analysis was performed to measure downstream PI3K. HFL1 cells were treated with TGF‑β1 (10 ng/ml) and LY294002 (10 μM) for 24 h. **E**–**H** A western blot analysis of the SREBP1, FASN, and SCD1 protein levels was performed. **I**–**K** qPCR was performed to measure the mRNA levels of ACACA, FASN, and SCD1. **L**–**O** The protein levels of fibronectin, COL1A1, and α-SMA were detected by western blotting. **P**–**S** The mRNA levels of FN1, COL1A1, COL3A1 and ACTA2 were measured by qPCR. The data are representative of three or six independent experiments and are presented as the means ± SEMs. *P < 0.05, **P < 0.01, ***P < 0.001, and ****P < 0.0001, as determined by one-way ANOVA with the Tukey‒Kramer post hoc test

Previous studies have shown that the PI3K-Akt-mTOR signaling pathway is activated by TGF-β and promotes protein synthesis and cell metabolism, which are required for cell growth, proliferation, and differentiation [22]. Thus, we treated HFL1 cells with TGF-β1 and the PI3K inhibitor LY294002. We found that treatment with LY294002 significantly inhibited the phosphorylation of Akt and its downstream effector molecule mTOR (Fig. 5A–D). Moreover, the inhibition of PI3K notably decreased the TGF-β1-induced upregulation of SREBP1, FASN, and SCD1 in HFL1 cells (Fig. 5E–H). Similarly, the transcript levels of the SREBP1 target genes ACACA, FASN, and SCD1 were reduced in HFL1 cells treated with TGF-β1 and LY294002 (Fig. 5I–K). In addition, the inhibition of PI3K decreased the TGF-β1-induced upregulation of fibronectin, COL1A1 and α-SMA in HFL1 cells (Fig. 5L–O). The mRNA levels of FN1, COL1A1, COL3A1, and ACTA2 in HFL1 cells were also significantly decreased by LY294002 (Fig. 5P–S). Taken together, these results demonstrate that TGF-β1 promotes SCD1 expression and fibroblast activation through PI3K-Akt-mTOR signaling.

### mTOR promotes SREBP1 nuclear localization, SCD1 expression, and fibroblast activation downstream of TGF-β1

**Fig. 6:**
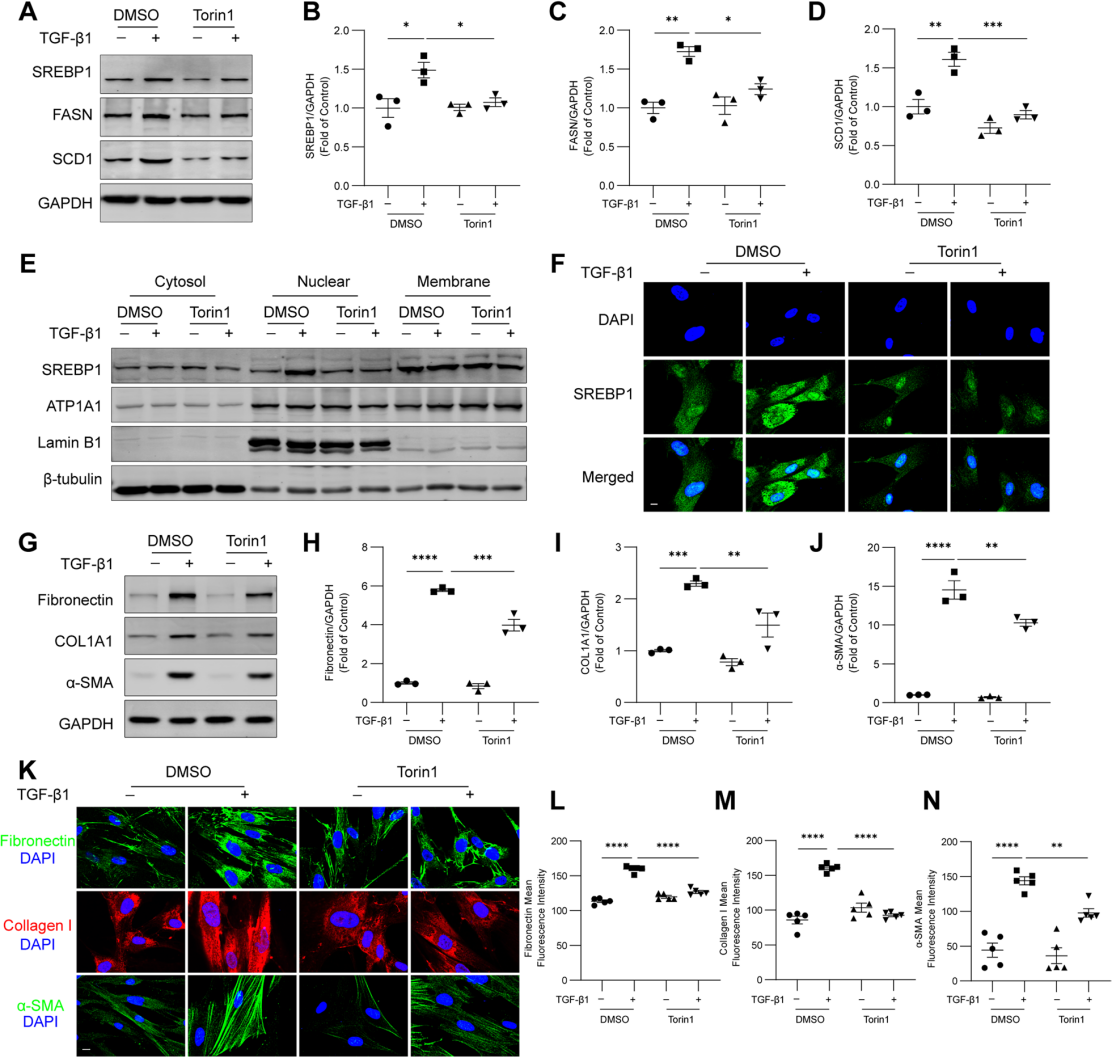
mTOR promotes SREBP1 nuclear localization, SCD1 expression, and fibroblast activation downstream of TGF-β1. HFL1 cells were treated with TGF‑β1 (10 ng/ml) and Torin1 (5 nM) for 24 h. **A**–**D** A western blot analysis of SREBP1, FASN and SCD1 in HFL1 cells was performed. **E** The protein levels of SREBP1 in cytosol, nuclear and membrane extracts were detected by western blotting. **F** The intracellular localization of SREBP1 was visualized by immunofluorescence. Scale bar, 10 μm. **G**–**J** The protein levels of fibronectin, COL1A1, and α-SMA were measured by western blotting. **K**–**N** The immunofluorescence staining of HFL1 cells with antibodies against fibronectin, collagen I, and α-SMA was captured by confocal microscopy. Scale bar, 10 μm. The fluorescence intensities were quantified (n = 5 images from each group were used for quantification). The data are representative of three independent experiments and are presented as the means ± SEMs. *P < 0.05, **P < 0.01, ***P < 0.001, and ****P < 0.0001, as determined by one-way ANOVA with the Tukey‒Kramer post hoc test

mTOR, a downstream effector of Akt activation, impacts SREBP1 transcription, processing and nuclear localization [23]. To further establish the role of mTOR in regulating fatty acid synthesis and fibroblast activation downstream of TGF-β1, we treated HFL1 cells with TGF-β1 and Torin1, an inhibitor of mTOR complexes. Treatment with Torin1 reduced the protein levels of SREBP1, FASN and SCD1 in HFL1 cells (Fig. 6A–D). Furthermore, treatment with Torin1 inhibited the nuclear localization of SREBP1 (Fig. 6E, F). The TGF-β1-induced expression of fibronectin, COL1A1, and α-SMA was also partially reduced by treatment with Torin1 (Fig. 6G–J). Immunofluorescence staining showed similar results: the protein expression of fibronectin, collagen I, and α-SMA in HFL1 cells was reduced by treatment with Torin1 (Fig. 6K–N). The above data demonstrate that mTOR regulates the TGF-β1-induced nuclear localization of SREBP1, expression of fatty acid synthesis enzymes, and fibroblast activation.

## Discussion

**Fig. 7:**
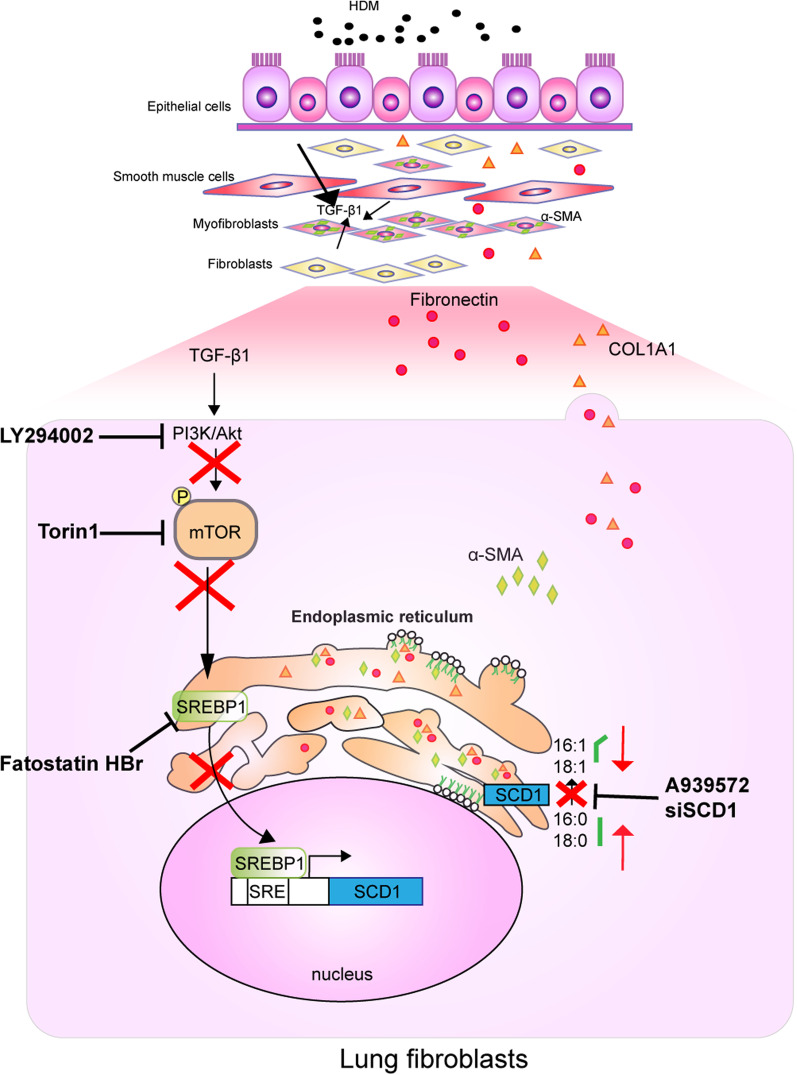
PI3K-Akt-mTOR-SREBP1 signaling regulates TGF-β1-induced SCD1 expression in lung fibroblasts. In lung fibroblasts, TGF-β1 promotes SCD1 expression via the PI3K-Akt-mTOR-SREBP1 signaling pathway. The downregulation of SCD1 expression decreases MUFAs and hinders high levels of ECM protein synthesis and transport in lung fibroblasts

In the present study, we demonstrated that SCD1 was essential for HDM-induced airway remodeling and TGF-β1-induced fibroblast activation in HFL1 cells. We further showed that TGF-β1 directly activated the PI3K-Akt-mTOR pathway to regulate SREBP1 nuclear localization and the subsequent induction of SCD1 (Fig. 7).

SCD1 has been extensively researched in lung cancer pathogenesis and is critical for cell proliferation and metastasis [24]. However, the role of SCD1 in chronic lung diseases remains unclear. In this study, we found that SCD1 inhibition effectively attenuated airway remodeling in an HDM-induced chronic asthma mouse model. Further investigation suggested that SCD1 inhibition reduced fibroblast activation in vivo.

Chronic airway inflammation is an important feature of asthma. Our results showed that SCD1 inhibition failed to reduce airway inflammation. This finding agrees with data reported by Rodriguez-Perez et al., who found a reduction in SCD1 in bronchial epithelial cells from murine models of allergic airway inflammation [9], and these findings suggest a possibility of airway inflammation-mediated SCD1 reduction in epithelial cells. Therefore, the administration of A939572 to mice exposed to HDM may further exacerbate the downregulation of SCD1 in epithelial cells. However, whether decreased expression of SCD1 in epithelial cells contributes to airway inflammatory responses should be explored further.

Fibroblast activation is an important mechanism associated with airway remodeling that contributes to cytoskeletal protein expression, ECM protein production, and collagen deposition. SCD1 has been implicated in the response of normal fibroblasts to growth factors, and the inhibition of SCD1 blunts the proliferation of fibroblasts and modulates membrane fluidity [25]. Our results demonstrated that SCD1 was upregulated by TGF-β1 in HFL1 cells. The inhibition or knockdown of SCD1 partially suppressed myofibroblast differentiation and ECM protein production induced by TGF-β1.

Previous studies have also shown that OA restores the proliferation of cancer cells after pharmacological inhibition of SCD1 [26, 27]. Thus, we exogenously added OA to HFL1 cells to observe whether OA could rescue the A939572-mediated inhibition of fibroblast activation in HFL1 cells. We found that exogenous OA alleviated the effects of A939572. These results may provide additional evidence for the role of SCD1 in profibrotic effects in not only asthma airway remodeling but also other fibrotic lung diseases. Elevated levels of MUFAs are vital for cells to maintain optimal membrane fluidity in response to increased protein and lipid trafficking [28]. However, we did not evaluate fatty acid levels, and further research should be performed using lipidomics tools to study the regulation of lipid metabolism upon fibroblast activation.

SREBP1 is a critical transcription factor that regulates de novo lipogenesis and lipid metabolism [21]. In our study, treatment with TGF-β1 resulted in increased SREBP1 expression, and SREBP1 inhibition significantly reduced TGF-β1-induced expression of SCD1 and fibroblast activation in HFL1 cells. This finding was consistent with previous studies demonstrating that SREBP inhibition blocked TGF-β1-induced upregulation of α-SMA and profibrogenic signaling in fibroblasts [29, 30]. Interestingly, SREBP1 is also activated by liver X receptor (LXR), which has been shown to reduce airway remodeling [31]. Indeed, the LXR ligand inhibited a-SMA and collagen in the lungs of an ovalbumin-induced mouse model of allergic asthma. Notably, the levels of TGF-β1 and MMP-9 were obviously reduced by pharmacologic activation of LXR, and LXR activation inhibited fibroblast activation and collagen release by interfering with the infiltration of macrophages and their release of profibrotic interleukin-6 [32], indicating that other signaling pathways associated with fibrosis could be affected by the agonist.

A recent study found that PI3K-Akt-mTOR signaling is involved in TGF-β-induced metabolic reprogramming in lung fibroblasts, as characterized by increased de novo synthesis of glycine and elevated levels of glycolysis and mitochondrial oxygen consumption [33]. We further investigated whether lipid metabolism is a target of mTOR signaling in TGF-β1-induced fibroblast activation. Our results clearly showed that TGF-β1 activated lipid synthesis-related enzymes and ECM protein production via the PI3K-Akt-mTOR signaling pathway.

## Conclusions

Here, we demonstrate that TGF-β1 activates SCD1 expression in lung fibroblasts via the PI3K-Akt-mTOR-SREBP1 pathway. These findings highlight the importance of lipid metabolism in the profibrotic effects on airway remodeling and suggest that the inhibition of SCD1 may be a novel therapeutic approach for relieving airway remodeling by modulating fibroblast activation.

## Supplementary Information

**Additional file 1: Figure S1. **Inhibition of SCD1 reduced HDM-induced fibroblast activation in vivo. **Figure S2.** OA is required for TGF-β1-induced fibroblast activation. **Table S1.** The siRNA sequence information. **Table S2.** The antibodies used in this study. **Table S3.** The primers used for qPCR analysis.

## Data Availability

The datasets used and/or analyzed during the current study are available from the corresponding author on reasonable request.

## Abbreviations

TGF-β1: Transforming growth factor-β1
SCD1: Stearoyl-CoA desaturase 1
PI3K: Phosphatidylinositol-3-kinase
Akt: AKT serine-threonine protein kinase
mTOR: Mechanistic target of rapamycin
SREBP1: Sterol regulatory element-binding protein 1
AHR: Airway hyper-responsiveness
α-SMA: Alpha-smooth muscle actin
ECM: Extracellular matrix
BALF: Bronchoalveolar lavage fluid
SFAs: Saturated fatty acids
MUFAs: Monounsaturated fatty acids
OA: Oleic acid
HDM: House dust mite
ELISA: Enzyme-linked immunosorbent assay
IgE: Immunoglobulin E
BSA: Bovine serum albumin
siSCD1: Small interfering RNA targeting SCD1
siNC: Negative control small interfering RNA
PBS: Phosphate-buffered saline
HE: Hematoxylin and eosin
PAS: Periodic acid-Schiff
IHC: Immunohistochemistry
qPCR: Quantitative real-time polymerase chain reaction
COL1A1: Collagen type I alpha 1
COL3A1: Collagen type III alpha 1
ACACA: Acetyl-CoA carboxylase alpha
FASN: Fatty acid synthase
LXR: Liver X receptor
