# Supplementary material for: TGF-β1 promotes SCD1 expression via the PI3K-Akt-mTOR-SREBP1 signaling pathway in lung fibroblasts
Source: Respir Res. 2023 Jan 10;24:8. doi: 10.1186/s12931-023-02313-9 (PMC9832654; doi:10.1186/s12931-023-02313-9)
Supplement: Supplementary file 1 — Additional file 1: Figure S1. Inhibition of SCD1 reduced HDM-induced fibroblast activation in vivo. Figure S2. OA is required for TGF-β1-induced fibroblast activation. Table S1. The siRNA sequence information. Table S2. The antibodies used in this study. Table S3. The primers used for qPCR analysis. [file 12931_2023_2313_MOESM1_ESM.docx]

**Additional files**

**Supplementary materials and methods**

**Reagents**

Enzyme-linked immunosorbent assay (ELISA) kits for TGF-β1 were purchased from R&D Systems (Minneapolis, MN, USA).

**Figure S1. Inhibition of SCD1 reduced HDM-induced fibroblast activation in vivo.**


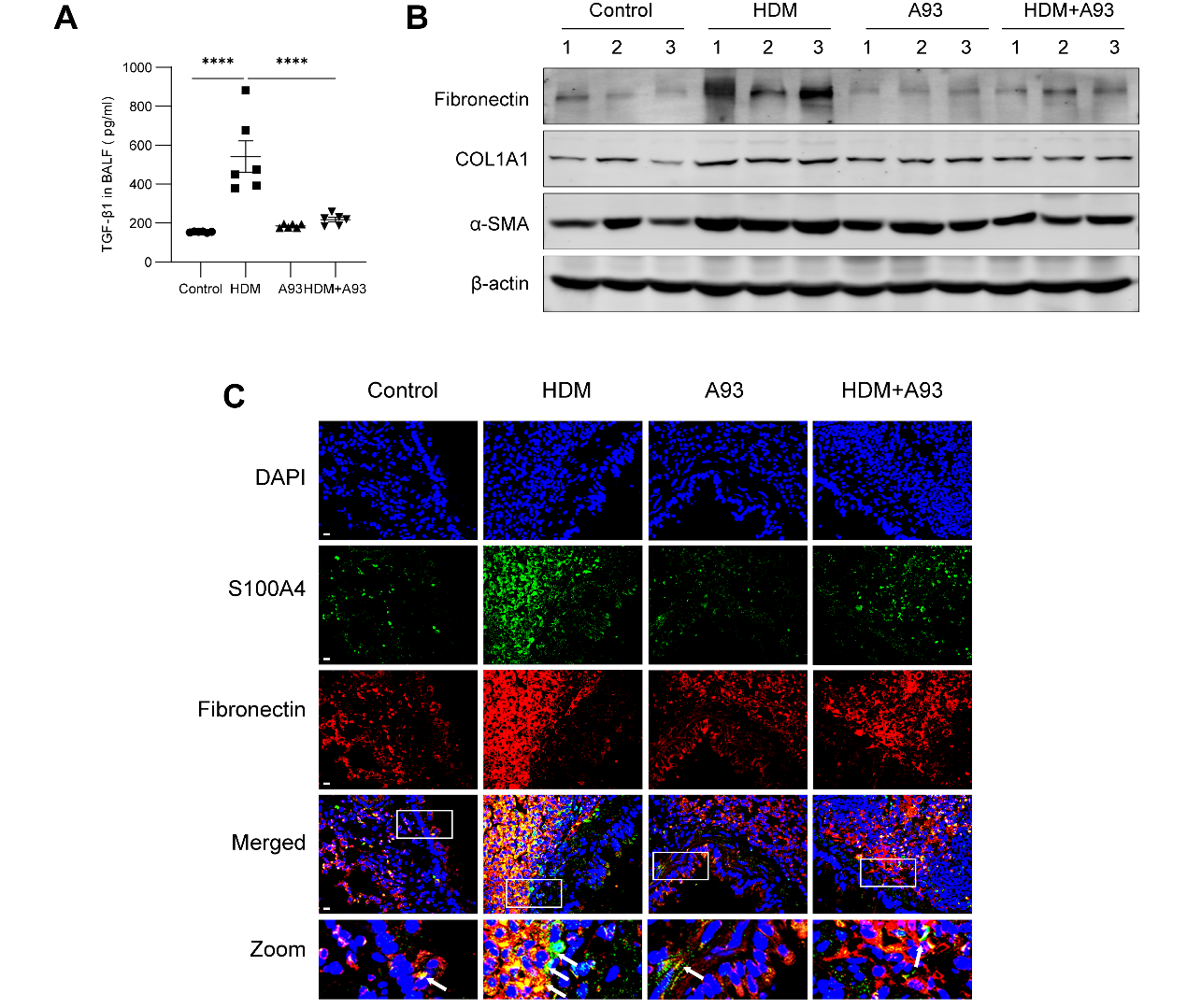


(A) The levels of TGF-β1 in BALF were measured by ELISA (n=6 mice in each group). (B) The protein levels of fibronectin, COL1A1 and α-SMA in lung homogenates were measured by western blotting. (C) Representative immunofluorescence colocalization images of S100A4 and fibronectin in the lung tissues from the different treatment groups, and DAPI was used to label nuclei. Scale bar, 10 μm. Statistical analysis was performed by one-way ANOVA with the Tukey‒Kramer post hoc test. **P*<0.05, ***P*<0.01, ****P*<0.001, and *****P*<0.0001.

**Figure S2.** **OA is required for TGF-β1-induced fibroblast activation.**


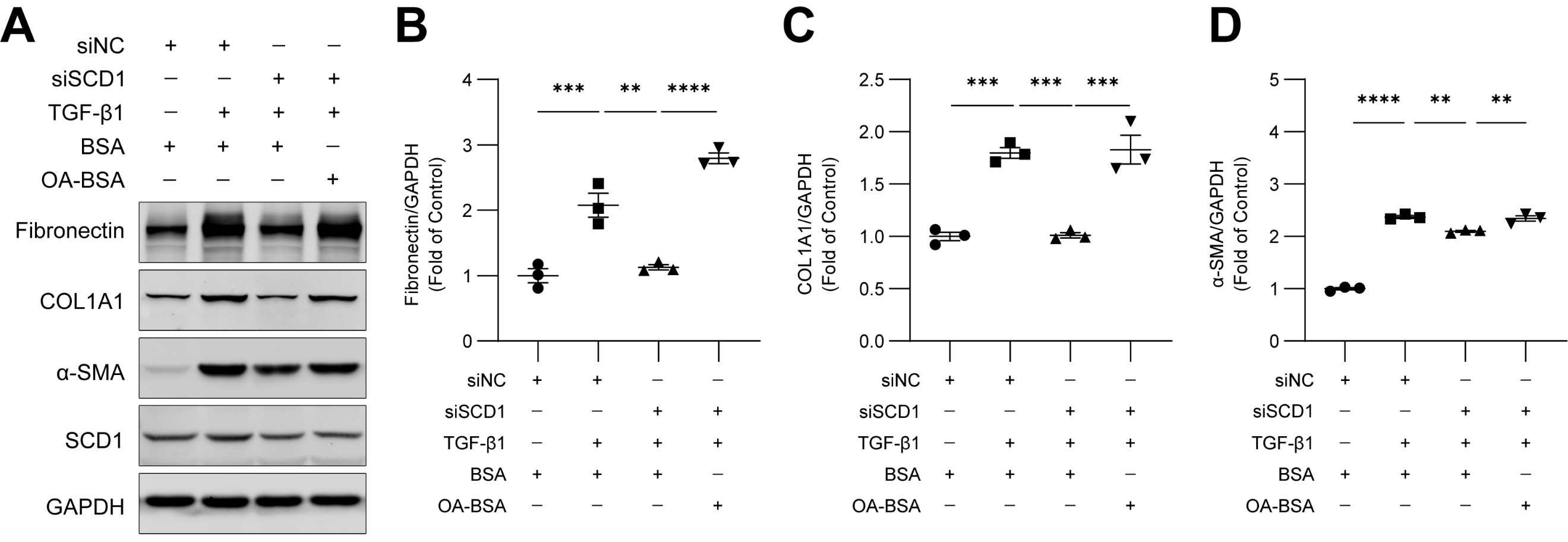


HFL1 cells were treated with siNC or siSCD1 for 48 h and then with TGF-β1 (10 ng/ml) and OA (0.3 mM) for 24 h. (A) The protein levels of fibronectin, COL1A1, α-SMA, and SCD1 were measured by western blotting. (B-D)The relative changes in band densities were detected. Data are representative of three independent experiments and are presented as the means ± SEMs. **P*<0.05, ***P*<0.01, ****P*<0.001, and *****P*<0.0001, as determined by one-way ANOVA with the Tukey‒Kramer post hoc test.

Table S1. The siRNA sequence information.

| siSCD1 | Sense(5'-3') | GGAGAAACAUCAUCCUUAUTT |
| --- | --- | --- |
| siSCD1 | Antisense(5'-3') | AUAAGGAUGAUGUUUCUCCTT |
| siNC | Sense(5'-3') | UUCUCCGAACGUGUCACGUTT |
| siNC | Antisense(5'-3') | ACGUGACACGUUCGGAGAATT |

Table S2. The antibodies used in this study.

| Antibodies | Source | Identifier |
| --- | --- | --- |
| Fibronectin | Abcam | ab2413 |
| COL1A1 | Abclonal | A1352 |
| Collagen Ⅰ | Abcam | ab34710 |
| α-SMA | Abcam | ab7817 |
| β-actin | Proteintech | 60008-1-Ig |
| S100A4 | NOVUS | 1F12-1G7 |
| SCD1 | Abclonal | A16429 |
| GAPDH | Proteintech | 60004-1-AP |
| SREBP1 | Santa cruz biotechnology | sc-365513 |
| SREBP1 | Abcam | ab28481 |
| FASN | Proteintech | 10624-2-AP |
| Akt | Cell Signaling Technology | 4685S |
| p-Akt(T308) | Cell Signaling Technology | 2965S |
| p-Akt(S473) | Cell Signaling Technology | 4060S |
| mTOR | Proteintech | 66888-1-Ig |
| p-mTOR(S2448) | Cell Signaling Technology | 5536S |
| ATP1A1 | Proteintech | 14418-1-AP |
| Lamin B1 | Proteintech | 66095-1-Ig |
| β-tubulin | Abclonal | AC021 |

Table S3. The primers used for qPCR analysis.

| FN1 Forward primer | AATAGATGCAACGATCAGGACA |
| --- | --- |
| FN1 Reverse primer | GCAGGTTTCCTCGATTATCCTT |
| COL1A1 Forward primer | AAAGATGGACTCAACGGTCTC |
| COL1A1 Reverse primer | CATCGTGAGCCTTCTCTTGAG |
| COL3A1 Forward primer | CTCAGGGTGTCAAGGGTGAAAGTG |
| COL3A1 Reverse primer | TGTACCAGCCAGACCAGGAAGAC |
| ACTA2 Forward primer | TCGTGCTGGACTCTGGAGATGG |
| ACTA2 Reverse primer | CCACGCTCAGTCAGGATCTTCATG |
| SCD1 Forward primer | CTTGCGATATGCTGTGGTGC |
| SCD1 Reverse primer | AAGTTGATGTGCCAGCGGTA |
| ACACA Forward primer | TACCTTCTTCTACTGGCGGCTGAG |
| ACACA Reverse primer | GCCTTCACTGTTCCTTCCACTTCC |
| FASN Forward primer | CCATCTACAACATCGACACCAG |
| FASN Reverse primer | CTTCCACACTATGCTCAGGTAG |
| GAPDH Forward primer | ATCAGCAATGCCTCCTGCAC |
| GAPDH Reverse primer | TGGCATGGACTGTGGTCATG |
